# Brain-derived neurotrophic factor receptor TrkB exists as a preformed dimer in living cells

**DOI:** 10.1186/1750-2187-7-2

**Published:** 2012-01-24

**Authors:** Jianying Shen, Ichiro N Maruyama

**Affiliations:** 1 Information Processing Biology Unit, Okinawa Institute of Science and Technology Graduate University, Okinawa 904-0495, Japan; 2 Department of Immunology, The Bechman Research Institute of the City of Hope, Duarte, CA 91010, USA

**Keywords:** BDNF, Chemical crosslinking, Protein fragment complementation assay, Neurotrophin receptor, Preformed homodimer

## Abstract

**Background:**

Neurotrophins (NTs) and their receptors play crucial roles in the development, functions and maintenance of nervous systems. It is widely believed that NT-induced dimerization of the receptors initiates the transmembrane signaling. However, it is still controversial whether the receptor molecule has a monomeric or dimeric structure on the cell surface before its ligand binding.

**Findings:**

Using chemical cross-linking, bimolecular fluorescence complementation (BiFC) and luciferase fragment complementation (LFC) assays, in this study, we show the brain-derived neurotrophic factor (BDNF) receptor TrkB exists as a homodimer before ligand binding. We have also found by using BiFC and LFC that the dimer forms in the endoplasmic reticulum (ER), and that the receptor lacking its intracellular domain cannot form the dimeric structure.

**Conclusions:**

Most, if not all, of the TrkB receptor has a preformed, yet inactive, homodimeric structure before BDNF binding. The intracellular domain of TrkB plays a crucial role in the spontaneous dimerization of the newly synthesized receptors, which occurs in ER. These findings provide new insight into an understanding of a molecular mechanism underlying transmembrane signaling mediated by NT receptors.

## Findings

The neurotrophin (NT) receptor family consists of the tropomyosin-related kinase receptors (Trk) A, B and C, and p75^TNR^, a member of the tumor necrosis factor (TNF) receptor superfamily. TrkA preferentially interacts with the nerve growth factor (NGF), TrkB with BDNF and NT-4/5, and TrkC with NT-3. NT-3 can also interact with TrkA and TrkB with relatively low affinity, and all the NTs can bind p75^NTR ^with low affinity [1,2]. The Trk receptor kinases play crucial roles in the development and maintenance of the central and peripheral nervous system, and consist of an extracellular ligand-binding domain, a single transmembrane domain and an intracellular tyrosine kinase domain. Upon activation, the receptor kinases initiate downstream signaling cascades mediated by Ras/Raf/MAP kinase, PI3K/Akt and PLC-γ [2-4]. TrkB and its ligand BDNF are highly expressed in biologically unfavourable neuroblastomas, and TrkB expression is associated with drug resistance and expression of angiogenic factors [5].

It is widely believed that the Trk receptor kinases are activated through NT-induced receptor dimerization. Because the NT exists in solution as a stable homodimer [6,7], it is thought that the NT dimer acts as a bridge to induce the dimerization of two Trk monomers. However, we have recently found that TrkA exists as a preformed, yet inactive, dimer in living cells [8]. In the present study, therefore, we also examined if TrkB exists as a preformed dimer in living cells before BDNF binding.

### Preformed TrkB homodimers detected by chemical crosslinking

**Figure 1:**
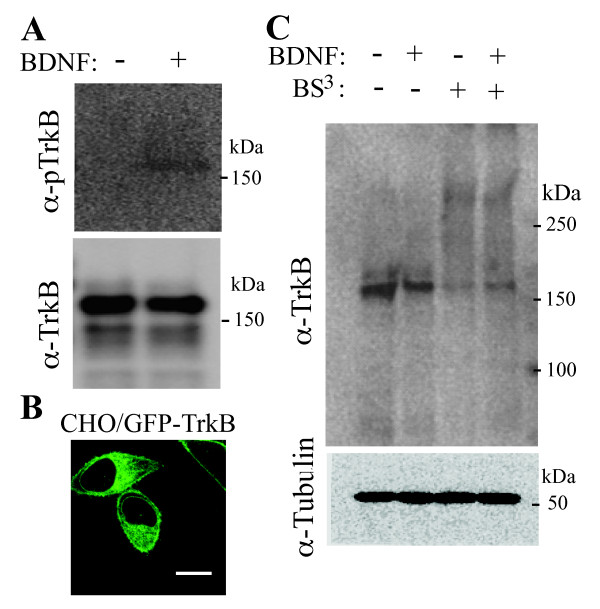
**Chemical crosslinking of TrkB on the cell surface**. **(A) **CHO cells expressing GFP-TrkB were stimulated with (+) or without (-) 100 ng/ml of BDNF for 5 min at 37°C. The cells were lysed and subjected to Western blotting with antibodies, α-pTrkB or α-TrkB, against phosphorylated TrkB or TrkB, respectively. Note that the fusion receptors were phosphorylated only in the presence of BDNF. **(B) **CHO cells expressing GFP-TrkB were serum-starved for 3 h as described in Methods. Fluorescence signal of GFP were observed by confocal microscopy. This figure represents > 10 similar images observed. Bars, 10 μm. **(C) **CHO cells expressing GFP-TrkB were serum-starved, and then were incubated with culture media containing 0.45 M sucrose for 20 min at 37°C. After being incubated in the presence (+) or absence (-) of 100 ng/ml of BDNF for 5 min at 37°C, the cells were treated with (+) or without (-) the chemical cross-linker BS^3 ^as described in Methods. The cells were lysed and subjected to Western blotting, with antibody against TrkB. This figure represents similar results of three independent experiments. Alpha-tubulin was visualized as a reference. The positions of molecular weight standards are shown at the right of each panel.

We first established a Chinese hamster ovary (CHO) cell line stably expressing green fluorescent protein (GFP)-fused TrkB at the receptor's N-terminus. The GFP-TrkB fusion protein was inactive in the absence of BDNF, and could be activated by BDNF added to the culture media, as shown by Western blotting using antibody against phosphorylated TrkB (Figure 1A). The cells were serum-starved for 2 h at 37°C, followed by an-hour incubation on ice, in order to increase the cell-surface expression of the receptor chimera (Figure 1B). Furthermore, sucrose, 0.45 M at a final concentration, was added to the culture media in order to inhibit the clathrin-dependent endocytosis of GFP-TrkB upon BDNF binding [9,10]. Then, the receptor molecules on the cell surface were chemically cross-linked with BS^3^, a membrane-impermeable cross-linker, in the presence or absence of BDNF. After the cross-linking, the cell lysates were subjected to Western blotting, and immunostained with anti-TrkB antibody. In the absence of the cross-linker, only the lower molecular mass band, ~170 kDa, was detected, while in the presence of the cross-linker higher molecular mass band, ~340 kDa, was also recognized by anti-TrkB antibody in addition to the lower band. Based on the apparent molecular weights, we assigned the lower and higher bands as the monomer and dimer of TrkB, respectively. The band intensity of the TrkB dimer cross-linked was similar in the absence and presence of BDNF (Figure 1C). These results suggest either that a fraction of TrkB exists as a preformed dimer, or that only a fraction of the receptor dimers was expressed on the cell surface and was chemically cross-linked. Furthermore, the results also indicate that the dimer fraction was not increased after being incubated with BDNF.

### Preformed TrkB homodimers detected by BiFC

**Figure 2:**
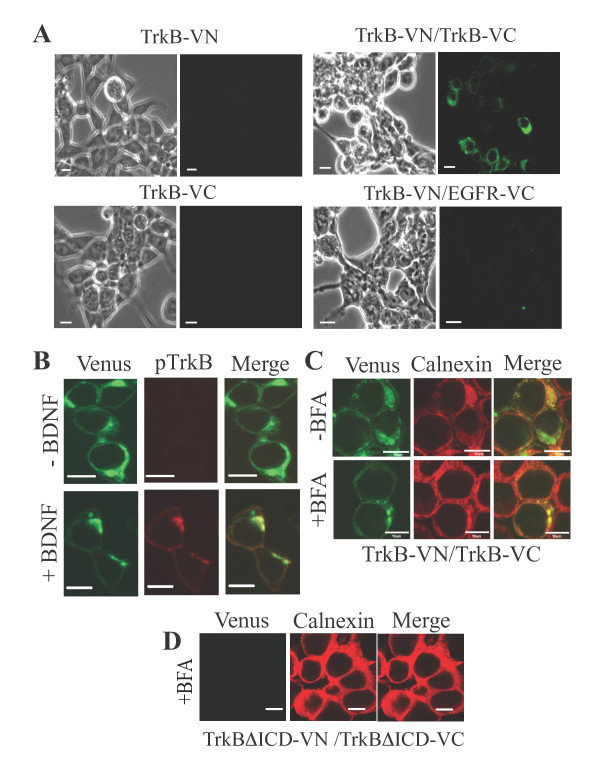
**BiFC assay of TrkB homodimers**. **(A) **HEK 293 cells were transfected with TrkB-VC alone, TrkB-VN alone, TrkB-VN and TrkB-VC, or TrkB-VN and EGFR-VC. Twenty-four hours after the transfection, Venus fluorescence signals were observed by confocal microscopy. This figure represents > 20 similar images observed. **(B) **HEK 293 cells co-expressing TrkB-VN and TrkB-VC were treated with (+) or without (-) 100 ng/ml of BDNF. The cells were fixed, and immunostained with anti-pTrkB primary antibody and then with fluorescently labeled secondary antibody. Fluorescence signals of Venus (green) and pTrkB (red) were observed by confocal microscopy. Note that the green and red fluorescence images do not completely overlap since TrkB-VN and TrkB-VC can form non-fluorescent homodimers. This figure represents > 20 similar images derived from four independent experiments. Bars, 10 μm. **(C, D) **HEK 293 cells co-expressing TrkB-VN and TrkB-VC **(C)**, or the fusion receptor pair lacking the intracellular domain, TrkBΔICD-VN and TrkBΔICD-VC **(D) **were treated with (+) or without (-) 10 μg/ml BFA. The cells were fixed, and immunostained with anti-calnexin antibody and then with fluorescently labeled secondary antibody. These figures represent 15 similar images derived from three independent experiments. Bars, 10 μm.

To directly observe spontaneous dimerization and subcellular localization of TrkB in living cells, we employed BiFC [11,12]. The N-terminal and C-terminal fragments of the Venus fluorescent protein [13], a derivative of yellow fluorescent protein, were fused to the C-terminus of TrkB, separately, to make TrkB-VN and TrkB-VC, respectively. When these constructs were expressed together in the human embryonic kidney HEK 293 cells, Venus fluorescence was clearly observed, whereas no fluorescence was observed in HEK 293 cells expressing either TrkB-VN or TrkB-VC, or in cells co-expressing TrkB-VN and the epidermal growth factor receptor (EGFR)-VC as a negative control (Figure 2A). To exclude again the possibility that the TrkB dimers were activated due to the BiFC fusion, we examined the receptor phosphorylation in the absence or presence of BDNF. In the absence of BDNF, only very low level of basic autophosphorylation of the fusion receptors was observed, and BDNF treatment could enhance the phospholylation of the fused TrkB receptor molecules (Figure 2B). Together with the results shown in Figure 1A, these results confirm that the BiFC fusion receptors behave like the wild-type receptor, and suggest again that TrkB exists as an inactive dimer, which can be activated by BDNF binding.

The Venus fluorescence was observed not only on the cell surface but also inside the cells (Figure 2A, B). To examine where the Venus reconstitution occurs during the receptor synthesis, we treated the cells co-expressing TrkB-VN and TrkB-VC with Brefeldin A (BFA), a lactone antibiotic that disassembles the Golgi apparatus and blocks anterograde transport of membrane proteins from ER to Golgi [14]. In the absence of BFA, Venus fluorescence was clearly observed both on the cell surface and inside the cells (Figure 2C, upper panels), while in the presence of BFA, Venus fluorescence was observed only inside the cells (Figure 2C, lower panels). The fluorescence inside the cells was mainly co-localized with calnexin, an ER marker [15]. These results indicate that TrkB spontaneously dimerizes in ER before the newly synthesized receptor reaches the cell surface. In contrast, no Venus fluorescence was observed in HEK 293 cells co-expressing TrkBΔICD-VN and TrkBΔICD-VC, both of which lack the intracellular domain (ICD) of TrkB, in the presence of BFA (Figure 2D). This confirms that BFA treatment itself does not induce the Venus reconstitution, and suggests that the intracellular domain plays a crucial role in the spontaneous dimerization of TrkB.

### Preformed TrkB homodimers detected by LFC

**Figure 3:**
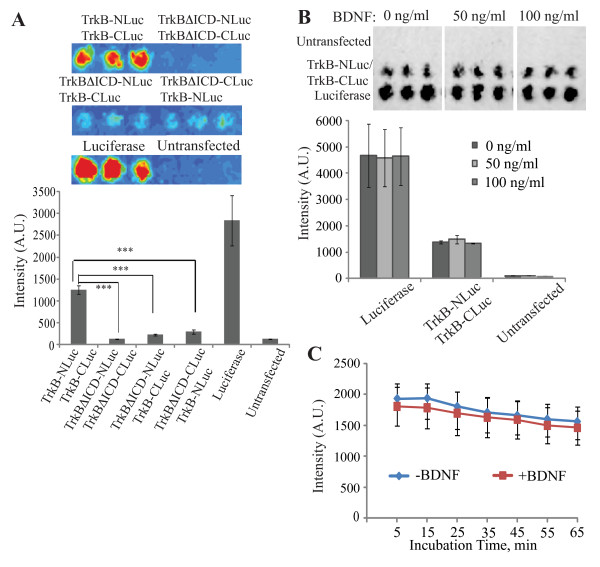
**LFC assay of TrkB homodimers**. **(A) **HEK 293 cells expressing indicated protein(s) were cultured in a microtiter plate, and then incubated with luciferin as described in Methods. The red color of the upper panels shows the intensity of bioluminescence produced by the cells in the microtiter wells, and the lower histogram shows the mean ± S.D. of triplicate experiments. *** *p *< 0.001. **(B) **HEK 293 cells expressing a full-length luciferase or co-expressing TrkB-NLuc and TrkB-CLuc were treated with BDNF at indicated final concentrations for 10 min, and then incubated with luciferin. The black color of the upper panels shows the intensity of bioluminescence produced by the cells in the micrtotiter wells. The lower histogram shows the mean intensity ± S.D. of triplicate experiments. **(C) **HEK 293 cells co-expressing TrkB-NLuc and TrkB-CLuc were stimulated with 100 ng/ml of BDNF in the presence of luciferin and 0.45 M sucrose to prevent endocytosis as described in Methods. Data are the mean ± S.D. of triplicate independent experiments.

Since the interaction of the BiFC Venus fragments is irreversible, we cannot determine the ratio of the TrkB dimer and monomer at equilibrium. Therefore, we employed firefly LFC because interaction of the luciferase fragments is reversible [16]. We fused the N-terminal or C-terminal fragment of firefly luciferase to the C-terminus of TrkB, resulting in TrkB-NLuc and TrkB-CLuc, respectively. When HEK 293 cells co-expressing TrkB-NLuc and TrkB-CLuc were incubated with luciferin in the absence of BDNF, bioluminescence was clearly detected although the bioluminescence level was lower than that of cells expressing pGL3, which encodes a full-length firefly luciferase (Figure 3A). In contrast, bioluminescence was not observed in cells co-expressing TrkBΔICD-NLuc and TrkBΔICD-CLuc, both of which lacked the intracellular domain of TrkB, in cells co-expressing TrkBΔICD-NLuc and TrkB-CLuc, or in cells co-expressing TrkB-NLuc and TrkBΔICD-CLuc. These results demonstrate that TrkB has a preformed homodimeric structure, and its intracellular domain plays a crucial role in the spontaneous dimerization as shown by the BiFC experiment above (Figure 2D). When the cells co-expressing TrkB-NLuc and TrkB-CLuc were treated with 50 or 100 ng/ml BDNF at a final concentration for 10 min at 37°C, the bioluminescence intensity produced by the cells was indistinguishable from that by cells untreated with BDNF (Figure 3B). Furthermore, Figure 3C shows that time courses of the bioluminescence intensity of cells co-expressing TrkB-NLuc and TrkB-CLuc were very similar each other in the presence or absence of 100 ng/ml BDNF. These results demonstrate that most, if not all, of TrkB on the cell surface has a homodimeric structure before BDNF binding.

In the present study, we have shown that TrkB exists as a homodimer in living cells before ligand binding, by using three different approaches, chemical cross-linking, BiFC and LFC. The results demonstrate that most, if not all, of the TrkB receptor molecules on the cell surface have homodimeric structures since the addition of BDNF to the culture media did not induce the dimerization at all (Figure 3B, C). The BiFC assay revealed that the spontaneous dimerization of TrkB occurs in ER before the newly synthesized receptors reach the cell surface (Figure 2C). The preformed TrkB homodimer is inactive since the receptor dimer was phosphorylated only when incubated with BDNF (Figures 1A and 2B). Furthermore, it has been demonstrated by both of the BiFC and LFC assays that the cytoplasmic domain of TrkB plays an important role in the spontaneous dimerization (Figures 2D and 3A).

An increasing number of studies demonstrate that many transmembrane receptors, which include receptors previously thought to be activated by ligand-induced receptor dimerization, exist as preformed, yet inactive, homo- and heterodimers in living cells. These receptors are the aspartate receptor Tar [17], epidermal growth factor receptor (EGFR) family [12,18-21], erythropoietin receptor [22], growth hormone receptor (GHR) [23,24], Toll-like receptor-9 [25], natriuretic peptide receptor A (NPRA) [26], and nerve growth factor receptor TrkA [8]. In the cases of EGFR, GHR, NPRA and Tar, it has been proposed that ligand binding is likely to induce the rotation/twist of the transmembrane domains of the preformed receptor dimers, resulting in rearrangement of the cytoplasmic domains for activation [18,24,26,27]. Therefore, further study of structures of the Trk receptor family will shed light on molecular mechanisms underlying the receptor activation.

## Methods

### Plasmid construction

BiFC vectors, pBiFC-VN173 and pBiFC-VC155, were described previously [28], and FLAG tags encoded by the vectors were removed as previously described [8]. PCR products that encode a full-length human TrkB protein were amplified using pLNCX-hTrkB [29], a generous gift from Dr. Garrett M. Brodeur (Children's Hospital of Philadelphia, PA), as a template and the following primer pairs: 5'-CCGgaattcACCATGTCGTCCTGGATAAGGTG (TrkB-VN173-F) and 5'-GCtctagaGCCTAGAATGTCCAGGTAGAC (TrkB-VN173-R), or 5'-CCGgaattcACCATGTCGTCCTGGATAAGGTG (TrkB-VC155-F) and 5'-CCGctcgagAGCCTAGAATGTCCAGGTAGAC (TrkB-VC155-R). The PCR products were inserted between *Eco *RI and *Xba *I sites of pBiFC-VN173, or between *Eco *RI and *Xho *I of pBiFC-VC155, resulting in pTrkB-VN or pTrkB-VC, respectively. Then, a synthetic double-stranded oligonucleotide, 5'-GGTGGTGGTGGTTCTGGTGGTGGTGGTTCTGGTGGTGGTGGTTCT encoding amino-acid residues GGGGSGGGGSGGGGS termed (G_4_S)_3_, were inserted into the unique *Xba *I or *Xho *I site of pTrkB-VN or pTrkB-VC, respectively. Plasmids for firefly (*Photinus pyralis*) LFC were described previously [8]. A DNA fragment encoding TrkB was cut out with *Eco *RI and *Xba *I enzymes from pTrkB-VN, and was then inserted into pBiFC-NLuc and pBiFC-CLuc, resulting in pTrkB-NLuc and pTrkB-CLuc, respectively. The construction of pGFP-TrkB was based on pEGFR-eGFP [30], and a DNA sequence encoding enhanced GFP, which was amplified by PCR using the primers 5'-CCGagatctATGGTGAGCAAGGGCGAGGAGCTGTTCACC (EGFP-F-BglII) and 5'-CCGgaattcCTTGTACAGCTCGTCCATGCCGAGAGTGATCC (EGFP-B-EcoRI), and was then inserted between *Bgl *II and *Eco *RI of pAEMXT-ACPwt-GPI (Covalys Bio-sciences, Witterswil, Switzerland), resulting in pAEMXT/GFP. A DNA sequence encoding human TrkB without its signal peptide (aa 1-31), but with (G_4_S)_3_, was inserted between *Eco *RI and *Xho *I of pAEMXT/GFP, resulting in pGFP-TrkB. To construct various plasmids encoding TrkB lacking its intracellular domain, a DNA fragment encoding the full-length TrkB of the expression plasmid constructs was replaced with a fragment encoding amino-acid residues 1-465 of TrkB. Validity of all the constructs was confirmed by DNA sequencing and Western blotting.

### Cell culture

A HEK 293 cell line was purchased from RIKEN cell bank (Cat. No.: RCB 1637; Ibaraki, Japan), and was maintained in Dulbecco's modified Eagle's medium (DMEM; Nacalai Tesque, Kyoto, Japan) supplemented with 10% fetal bovine serum (FBS; Invitrogen). A CHO-K1 cell line purchased from the American Type Culture Collection (ATCC; Manassas, VA) was maintained in Iscove's modified Dulbecco's medium (IMDM; Invitrogen). All media were supplemented with 100 U/ml of penicillin-streptomycin (Nacalai Tesque). A CHO-K1 cell line stably expressing the fusion GFP-TrkB was established by transfecting CHO-K1 cells with the pGFP-TrkB plasmid, followed by selection with 1.0 mg/ml of G418 (Nacalai Tesque). The established cells were treated with (+) or without (-) 10 μg/ml BFA for 12 h at 37°C.

### Chemical crosslinking

CHO cells expressing the fusion construct GFP-TrkB were cultured to a concentration of ~10^7 ^cells/dish (10 cm in diameter; Iwaki Asahi Techno Glass, Chiba, Japan) before chemical crosslinking. The cells were serum-starved for 2 h at 37°C, incubated on ice for another 1.0 h, and then incubated in IMDM containing 0.45 M sucrose for 20 min at 37°C. In the presence of 0.45 M sucrose, the cells were treated with or without 100 ng/ml of BDNF (Alomone Labs, Jerusalem, Israel) at a final concentration for 5 min at 37°C. Then, the cells were treated with or without 1.0 mM BS^3 ^(Pierce Biotechnology, Rochford, IL) at a final concentration in Dulbecco's phosphate-buffered saline (DPBS; Invitrogen), which contained 0.45 M sucrose, for 20 min at 37°C. This crosslinking reaction was quenched by adding 40 mM Tris-HCl, pH 8.0, and 0.45 M sucrose at final concentrations, followed by incubation for 15 min at room temperature. After being washed twice with ice-cold DPBS, the cells were lysed with NP-40 lysis buffer (50 mM Tris-HCl, pH 7.4, 250 mM NaCl, 5 mM EDTA, 50 mM NaF, 1.0 mM Na_3_VO_4_, 1.0% Nonidet P-40) supplemented with a protease inhibitor cocktail (Nakalai Tesque) and a phosphatase inhibitor cocktail (Nacalai Tesque) for 30 min at 4°C. Resulting cell lysates were sonicated twice for 5 s each, and centrifuged at 10,000 rpm for 10 min at 4°C. The supernatants were collected for Western blotting.

### Western blotting

Cell lysates, 10 μg each as the amount of total protein per lane, were loaded to NuPAGE 4-12% Bis-Tris gels (Invitrogen), followed by electrophoresis using XCell SureLock Mini-Cell (Invitrogen) and transferred onto Hybond-P PVDF membrane (Amersham Bioscences, Little Chalfont, UK) using a mini Trans-Blot electrophoretic transfer cell (Bio-Rad). The membrane was blocked overnight at 4°C in TBST (10 mM Tris-HCl, pH7.6, 0.9% NaCl, 0.1% Tween 20) supplemented with 5% skimmed milk (Wako Pure Chemical, Osaka, Japan) or in TBST supplemented with 2% ECL advance blocking agent (GE Healthcare, Little Chalfont, UK), and was then incubated with the following primary antibody in blocking buffer for 2 h at room temperature: anti-TrkB monoclonal antibody (mAb) (1:500 dilution; Novus Biologicals, Littleton, CO), anti-phospho-TrkA (Tyr674/675)/TrkB (Tyr706/707) (C50F3) rabbit mAb (1:000 dilution; Cell Signaling Technology, Tokyo, Japan), or mouse mAb against α-tubulin (1:2000 dilution; Sigma-Aldrich Japan, Tokyo). The membrane was washed three times with TBST, and was then incubated with sheep anti-mouse or donkey anti-rabbit secondary antibody conjugated with horseradish peroxidase (1:10000 dilution; GE Healthcare) in the same blocking mixture for 90 min at room temperature. After being washed four times with TBST, the membrane was reacted with an ECL advance Western blotting detection kit (GE Healthcare). Protein bands were visualized using a lumino-image analyser (LAS3000; Fujifilm, Tokyo, Japan).

### Immunofluorescent staining

Immunofluorescent staining was performed as previously described [12]. HEK 293 cells (~2 × 10^4 ^cells/well) were seeded into an eight-well Lab-Tek II chamber slide (Nalge Nunc International, Naperville, IL), and then transfected with or without expression plasmids of interest. After washing twice with ice-cold 1 × PBS, the cells were fixed with methanol-acetone (1:1) for 10 min at -20°C. After being blocked with a mixture of Blocking One-P (Nakalai Tesque) and 2% ECL Advance Blocking Agent (GE Healthcare) and 50 mM NaF for 1.0 h at room temperature, the cells were incubated with the following primary antibodies diluted in blocking buffer for 1.0 h at room temperature: rabbit mAb against phospho-TrkA (Tyr490)/TrkB (Tyr516) (Cell Signaling Technology) and rabbit anti-calnexin antibody (Sigma). After washed three times with TBST, the cells were incubated with the following secondary antibodies diluted in blocking buffer for 1.0 h at room temperature: Alexa Fluor 633-conjugated goat anti-rabbit IgG (H+L) antibody (Molecular Probes, Eugene, OR). After washed three times with TBST, the cells were mounted in Vectashield (Vector Laboratories, Burlingame, CA) and observed under an Axiovert 200 M inverted microscope equipped with an LSM 510 META Ver 3.5 scan head (Carl Zeiss, Jena, Germany), using 488 nm (argon) and 633 nm (HeNe) (Titanium:Sapphire from Mai Tai; Spectra-Physics, Mountain View, CA) laser lines for excitation of Venus and Alexa Fluor 633, respectively.

### Firefly LFC

HEK 293 cells were plated in a 96-well black polystyrene borosilicate glass microplate (Iwaki) at a concentration of ~8,000 cells/well 12 h before transfection. Then the cells were transfected with 50 ng of the control vector pGL3 (Promega, Madison, WI), which encoded a full-length luciferase, or with 100 ng each of expression plasmid constructs by using 0.1 μl of Plus Reagent (Invitrogen) and 0.25 μl of Lipofectamin LTX Reagent (Invitrogen) in 20 μl of Opti-MEM I reduced serum medium (Invitrogen). Forty hours after transfection, the cells were serum-starved for 3 h, washed once with DPBS, and then incubated in 100 μl of DPBS, which contained 0.15 mg/ml D-luciferin (Promega) as well as 0 ng/ml, 50 ng/ml or 100 ng/ml BDNF at a final concentration, for 10 min at room temperature. After being incubated in 50 μl DPBS containing 0.45 M sucrose for 10 min at room temperature, alternatively, the transfected cells were incubated with 50 μl DPBS, which contained 0.45 M sucrose and 0.15 mg/ml D-luciferin at final concentrations, in the presence or absence of 100 ng/ml BDNF at a final concentration at 37°C. Then, bioluminescence intensity was measured by using a LAS 3000 or a microplate luminometer, LB 960 Centro (Berthold Japan, Osaka). Data are presented as the mean ± S.D. of triplicate measurements. Statistical significance was assessed using Student's *t*-test. The *P *values less than 0.01 were assessed to be statistically significant.

## List of abbreviations

BDNF: Brain-derived neurotrophic factor; BFA: Brefeldin A; BiFC: bimolecular fluorescence complementation; DPBS: Dulbecco's phosphate-buffered saline; EGFR: epidermal growth factor receptor; ER: endoplasmic reticulum; GFP: green fluorescent protein; ICD: intracellular domain; LFC: luciferase fragment complementation; mAb: monoclonal antibody; NT: neurotrophin: Trk: tropomyosin-related kinase.

## Competing interests

The authors declare that they have no competing interests.

## Authors' contributions

The project was initiated by J.S. and I.N.M. All the experiments were designed by J.S. and I.N.M., and were carried out by J.S. The manuscript was written by J.S. and I.N.M. All authors read and approved the final manuscript.
